# E2F3 upregulation promotes tumor malignancy through the transcriptional activation of HIF-2α in clear cell renal cell carcinoma

**DOI:** 10.18632/oncotarget.10568

**Published:** 2016-07-13

**Authors:** Yu Gao, Hongzhao Li, Xin Ma, Yang Fan, Dong Ni, Yu Zhang, Qingbo Huang, Kan Liu, Xintao Li, Lei Wang, Yuanxin Yao, Qing Ai, Xu Zhang

**Affiliations:** ^1^ Department of Urology, Chinese PLA General Hospital/Chinese PLA Medical School, Beijing, 100853, P. R. China; ^2^ State Key Laboratory of Kidney Diseases, Chinese PLA General Hospital/Chinese PLA Medical School, Beijing, 100853, P. R. China

**Keywords:** clear cell renal cell carcinoma, E2F3, hypoxia-inducible factor-2, transcriptional regulation, carcinogenesis

## Abstract

The E2F3 transcriptional regulatory pathway plays a major part in multiple-cancer progression, but the specific contributions of this pathway to tumor formation and the progression of clear cell renal cell carcinoma (ccRCC) are not fully understood. Clinically, we demonstrated that E2F3 was overexpressed in advanced tumor features. Moreover, cytoplasmic restoration predicted the poor overall survival of ccRCC patients. As a remarkable oncogene for ccRCC, high HIF-2α levels closely correlated with E2F3 upregulation. We observed *in vitro* that E2F3 overexpression and knockdown regulated HIF-2α expression. Furthermore, we found that HIF-2α harbored multiple E2F3 binding sites in the promoters. Mechanistically, E2F3 acted to transactivate HIF-2α transcription, which in turn exerted a serial effect on the pivotal epithelial–mesenchymal transition-related genes. The RNA interference-mediated silencing of HIF-2α attenuated E2F3-enhanced cell migration and invasion *in vitro* and *in vivo*. Overall, our results identified HIF-2α as a direct target gene for E2F3 upregulation, which was critical for carcinogenesis and progression of ccRCC. Thus, targeting the E2F3–HIF-2α interaction may be a promising approach to ccRCC treatment.

## INTRODUCTION

Renal cell carcinoma (RCC), whose most common subtype is clears cell, is a urologic malignant neoplasm that may be fatal [1]. Few clinical measures are effective for late stage clear cell renal cell carcinoma (ccRCC), and the most widely one is biological targeted therapy [2]. The outcome of patients with high clinical stage and metastatic RCC (mRCC) is inevitably poor [3]. Therefore, the molecular mechanism underlying the pathogenesis of ccRCC needs to be explored.

Our previous findings showed that E2F1 was upregulated in ccRCC and may act as an important driver for ccRCC malignancy and progression [4]. E2F3 and other E2F transcription factors are the prevalent regulators of various genes. Dissociated with Rb protein, E2Fs1-3 exert their functions in the way of transcriptional activation [5]. It has been reported to involve in cell proliferation and migration in numerous instances, including human ovarian cancer, gastric cancer, and hepatocellular carcinoma [6–9]. However, the full mode of action of E2F3 in ccRCC has not yet been characterized.

Hypoxia inducible factor-α (HIF-2α) is a transcription factor degraded by the von Hippel Lindau (VHL)–ubiquitin ligase complex [10]. HIF-1α and HIF-2α are well known for their contrasting and cooperative properties in VHL-deficient gene-expressed ccRCC [11]. Recently, many studies have proven that the key regulator HIF-2α rather than HIF-2α alone was responsible for ccRCC tumorigenesis [12–14]. The VHL tumor suppressor protein targets HIF-α subunits for ubiquitin-mediated proteolysis [15]. Thus, in cells with inactivating mutations of both VHL alleles, HIF-α subunits are stabilized at high levels irrespective of cellular hypoxia [16]. It is well known that VHL is highly mutated in sporatdic ccRCC and there are also lots of studies reporting that VHL inactivation in ccRCC leads to HIF up-regulation. In addition, VHL inactivation in ccRCC is not only through mutation, but also though promoter methylation. In our further study, we found that HIF-2α had distinguished expressions in different ccRCC cell lines with non-functional VHL. So we hypothesized that certain modulation of HIF-2α may exist independent of VHL-HIF-2α axis.

A close link was established between E2F3 and HIF-2α protein expression levels in ccRCC samples. Meanwhile, the presence of certain putative binding sites for E2F3 was predicted in HIF-2α promoter. In a further study, the investigation into the mechanism of aberrant HIF-2α expression in ccRCC was conducted without VHL inactivation. We focused on the role of E2F3 and HIF-2α in ccRCC carcinogenesis *in vitro*, as well as on the subcellular localization of E2F3 in cancer tissues and its clinical significance. Moreover, we assessed the combined effects of E2F3 and HIF-2α on the proliferation, cell cycle, migration, and invasion of RCC cells both *in vitro* and *in vivo*.

## RESULTS

### Upregulation of E2F3 in ccRCC tissues and its correlation with clinical features

**Figure 1:**
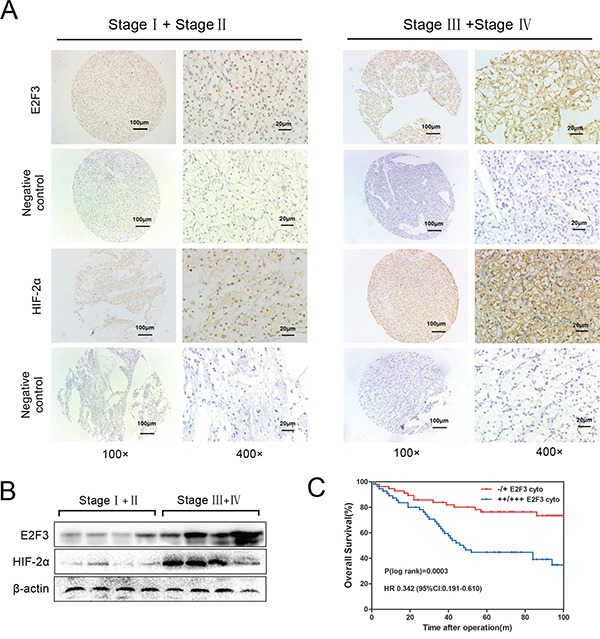
Upregulation of cytoplasmic E2F3 in ccRCC tissues **A**. ccRCC tissues (112 cases) of different clinical stages were immunohistochemically stained by E2F3 and HIF-2α antibody (1:100). Three representative photographs were taken at different magnifications in ccRCC tissues (100× and 400×). **B**. Protein expressions of E2F3 and HIF-2α were determined by western blot assay in patients with RCC. **C**. The overall survival of ccRCC patients calculated using Kaplan–Meier analysis according to low and high cytoplasmic E2F3 staining. High E2F3 protein expression was determined above the median H-score values. The P values were calculated using log-rank test.

Tissue microarray analysis from cohort patient specimen (112 cases) revealed the upregulation of cytoplasmic E2F3 gene in developmental stages, consistent with the differentiated protein expression result identified by western blot assay (Figure 1A and 1B). In addition, the normal kidney tissues were stained (Figure S1). Furthermore, we evaluated the correlation between E2F3 expression levels and the clinicopathological features of ccRCC patients in subcellular localization (Table 1). Among the 112 cancer specimens, elevated E2F3 immunostaining was observed in 48.2% (54 of 112) cytoplasm and 55.3% (62 of 112) nuclei. No significant correlation was found between the expression levels of E2F3 in age and gender. However, cytoplasmic and nuclear E2F3 expressions were correlated strongly with the tumor sizes (*p*=0.021 and *p*<0.001), clinical stages (*p*=0.030 and *p*=0.003), MVI (*p*=0.002 and *p*<0.001), and metastatic state (*p*=0.001 and *p*=0.016), except for histological grade (Fuhrman grade) (*p*=0.385 and *p*=0.028). High E2F3 nuclear (++ and +++) expression level was associated with smaller tumor sizes, lower clinical stages, incidence of microvascular invasion, and no metastatic state; whereas tumors with high cytoplasmic E2F3 (++ and +++) were positively associated with aggressive characteristics (Table 1). To further examine this clinical significance, Kaplan-Meier analysis was used to evaluate the overall survival among the cohorts of ccRCC patients. High E2F3 protein expression level was determined above the median H-score values. Strikingly, as Figure 1C showed, patients with low E2F3 cytoplasmic expression levels had significantly higher overall survival rates than those with high E2F3 expression (*p* = 0.0003; HR 0.342(95%CI:0.191-0.610)).

**Table 1:** Patient and tumor characteristics according to subcellular localisation of E_2_F_3_

Features		E_2_F_3_ nuclear immunostaining			E_2_F_3_ cytoplasm immunostaining		
		**-/+**	**++/+++**	***p*-value**	**-/+**	**++/+++**	***p*-value**
Age(y)							
≤60	77	36 (72.0%)	41 (66.1%)	0.505	37 (63.8%)	40 (74.1%)	0.241
>60	35	14 (28.0%)	21 (33.9%)		21 (35.1%)	14 (27.3%)	
Gender							
Male	75	32 (64.0%)	43 (69.4%)	0.549	42 (72.4%)	33 (61.1%)	0.204
Female	37	18 (36.0%)	19 (30.6%)		16 (27.6%)	21 (34.4%)	
Size(cm)							
≤7	52	13 (26.0%)	39 (62.9%)	<0.001*	33 (56.9%)	19 (35.2%)	0.021*
>7	60	37 (74.0%)	23 (37.1%)		25 (43.1%)	35 (64.8%)	
Stage							
Stage I	42	11 (22.0%)	31 (50.0%)	0.003*	28 (48.3%)	14 (25.9%)	0.030*
Stage II	29	12 (24.0%)	17 (27.4%)		16 (27.6%)	13 (24.1%)	
Stage III	26	16 (32.0%)	10 (16.1%)		9 (15.5%)	17 (31.5%)	
Stage IV	15	11 (22.0%)	4 (6.5%)		5 (8.6%)	10 (18.5%)	
Fuhrman grade							
G1/G2	81	31 (62.0%)	50 (80.6%)	0.028*	44 (75.9%)	37 (68.5%)	0.385
G3/G4	31	19 (38.0%)	12 (19.4%)		14 (24.1%)	17 (31.5%)	
MVI							
No	83	28 (56.0%)	55 (88.7%)	<0.001*	50 (86.2%)	33 (61.1%)	0.002*
Yes	29	22 (44.0%)	7 (11.3%)		8 (13.8%)	21 (38.9%)	
Metastasis							
No	97	39 (78.0%)	58 (93.5%)	0.016*	56 (96.5%)	41 (75.9%)	0.001*
Yes	15	11 (22.0%)	4 (6.5%)		2 (3.5%)	13 (24.1%)	

Clinical staging: Clinical stage grouping was assigned according to the 2009 TNM staging classification system. MVI (Microvascular invasion): small vessel invasion in ccRCC which indicated tumor aggressiveness.

### E2F3 regulates expression of HIF-2α

**Figure 2:**
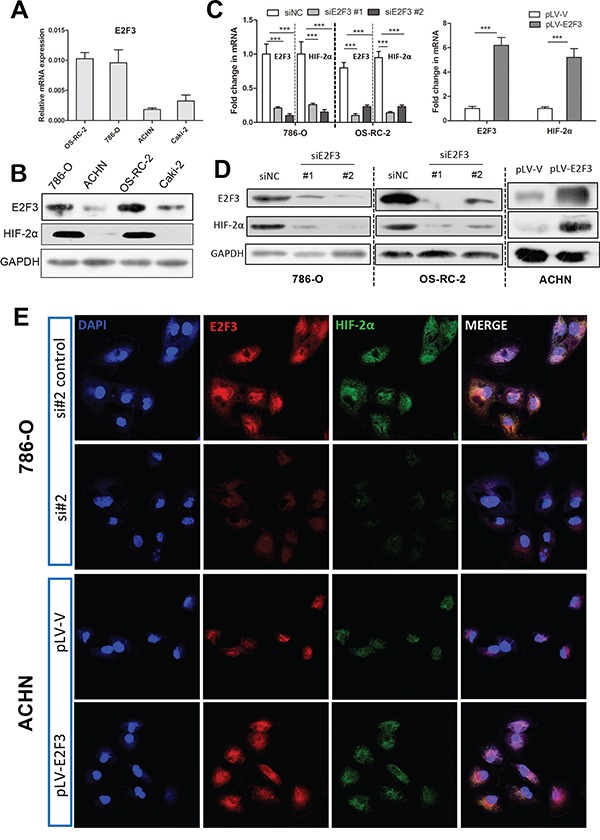
E2F3 regulates expression of HIF-2α **A**. Relative E2F3 mRNA levels in different ccRCC cell lines. **B**. Relative E2F3 and HIF-2α protein levels in different ccRCC cell lines. Glyceraldehyde-3-phosphate dehydrogenase (GAPDH) was used as internal control for equal loading of samples. **C**. Correspondent mRNA level changes of E2F3 and HIF-2α in 786-O and OS-RC-2 after E2F3 knockdown (left), the fold change in mRNA level of ACHN after E2F3 overexpression (right). **D**. Correspondent protein levels of E2F3 and HIF-2α were altered in 786-O and OS-RC-2 after E2F3 knockdown and in ACHN after E2F3 overexpression. **E**. Immunofluorescent staining was used to evaluate the protein expressions of E2F3 and HIF-2α after E2F3 knockdown and upregulation in different cell lines. E2F3 (red), HIF-2α (green), and 4′6-diamidino-2-phenylindole (DAPI, blue) (magnification, 600×) (* *p* <0.05, ** *p* <0.01, *** *p*<0.001).

To ascertain whether HIF-2α could be regulated by E2F3, we inhibited and overexpressed E2F3 in different cancer cell lines. Real-time and Western blot tests were applied in human renal cancer cell lines ranged from low in ACHN cells to high in OS-RC-2 and 786-O cells, depending on endogenous E2F3 protein levels (Figures 2A and 2B). We explored the influence of E2F3 transcriptional activity on the HIF-2α expression in cancer cells by using real-time techniques. Consistent with the mRNA level data, depletion of E2F3 severely impaired HIF-2α expression in tumor cells 786-O and OS-RC-2 transfected with siE2F3 (Figures 2C and 2D). Likewise, the transcript and protein levels of HIF-2α increased in ACHN upon E2F3 activation. The above data implied that E2F3 is essential for cancer cells to promote HIF-2α expression. In subsequent functional studies, the use of specific siRNA for different cell lines depended on the knockdown efficiency of E2F3. The findings were verified by real-time PCR and Western blot assay. The link between E2F3 and HIF-2α expression was further substantiated by confocal microscopy on 786-O, OS-RC-2, and ACHN. E2F3 and HIF-2α colocalized in the nuclei and the cytoplasm. HIF-2α staining immunofluorencence was abolished and upregulated in E2F3-silenced and overexpressed cell lines (Figure 2E).

### E2F3 binds and activates HIF-2α gene promoters

**Figure 3:**
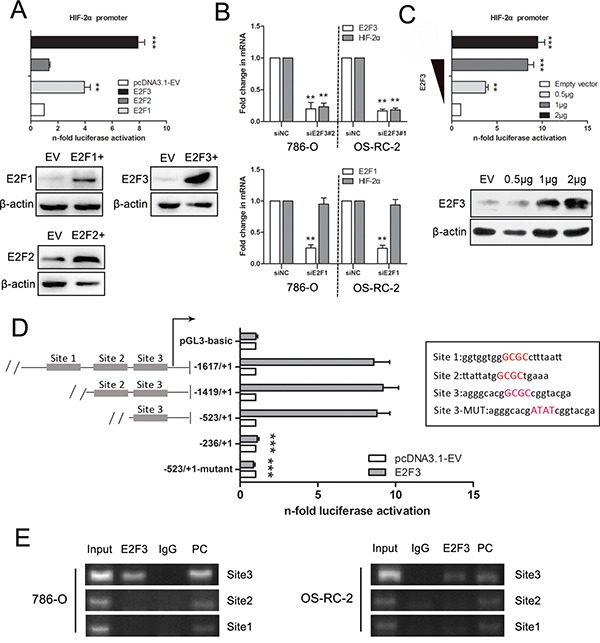
Luciferase and ChIP-PCR assay demonstrate the binding of E2F3 to the HIF-2α promoter in ccRCC cells **A**. Luciferase activities were measured in 293T cells after cotransfection of HIF-2α (-1617/+1) promoter reporter and different E2F family members. E2F1, E2F2, and E2F3 overexpression levels were verified by Western blot analysis. **B**. HIF-2α expression levels were examined after E2F1 and E2F3 knockdown in 786-O and OS-RC-2 cell lines. **C**. Relative luciferase activities were measured after cotransfection of promoter construct with increasing amounts (0.5, 1, and 2 μg) of E2F3 expression plasmid. Western blots confirm E2F3 protein expression after transfection. Actin was used for equal loading. **D**. Scheme of serially truncated and mutated HIF-2α promoter constructs along with the pRL-TK and E2F3 expression plasmids were transfected into 293T cells, and the quantification of firefly and *R. reniformis* activities was measured. The nucleotide sequences in the right panel represented the predicted binding sequences, with the red capital letters signifying core binding elements. The mutant sequences were also included. **E**. ChIP assay was performed in 786-O and OS-RC-2 cells using anti-E2F, normal rabbit IgG, and positive control. Input of sheared chromatin was prepared prior to immunoprecipitation. (*p<0.05, **p<0.01, ***p<0.001).

To determine whether the three E2F transactivators are able to upregulate HIF-2α, luciferase assays were performed after cotransfection of E2F1, E2F2, and E2F3 expression vectors and HIF-2α promoter reporter constructs. The results demonstrated that the promoter of HIF-2α was evidently activated by E2F1 and E2F3, whereas E2F2 had no effect. However, E2F3 seemed to be the primary activator of promoter HIF-2α. E2F1, E2F2, and E2F3 overexpression were verified by Western blot analysis (Figure 3A).

To ascertain the effect of E2F3 and E2F1 on HIF-2α expression, the mRNA level of HIF-2α was evaluated after the knockdown of E2F1 and E2F3 in cell lines. On the one hand, the decreased level of E2F3 resulted in a dramatic change of HIF-2α. On the other hand, HIF-2α appeared not to be affected by E2F1 regulation (Figure 3B). As shown in Figure 3C, the HIF-2α promoter vector (-1617/+1) was stimulated through E2F3 in a dose-dependent manner, which was in clear contrast to the control. Furthermore, analysis of the sequence upstream of the transcriptional initiation site by Genomatix(www.genomatix.de/en/index.html) revealed putative E2F3 binding sites at -1518/-1498 (Site 1), -1259/-1247 (Site 2), and -423/-403 (Site 3). 293T cells transfected with HIF-2α promoter fragment (-1617/+1) showed clear induction of luciferase activity with increasing amounts of E2F3. Truncation from -1617 to -1419 did not significantly affect promoter activation by cotransfected E2F3, as well as the segmental part of -1419 to -523; however, the DNA binding-mutant of Site 3 sequence had no stimulating effect (Figure 3D). The nucleotide sequences of the predicted binding sequences are listed in the right panel of Figure 3D with the red capital letters signifying core binding elements. The implication was that HIF-2α upregulation mainly occurs through site 3 (Figure 3D). ChIP was introduced to verify the responses of the three sites, and the results revealed a strong interaction between E2F3 and the motifs of HIF-2α located in Site 3 compared with the PC (positive control) and IgG (negative control) (Figure 3E).

### E2F3 protein promotes the proliferation of ccRCC cell lines and enhances the number of colony formation through HIF-2α activation

**Figure 4:**
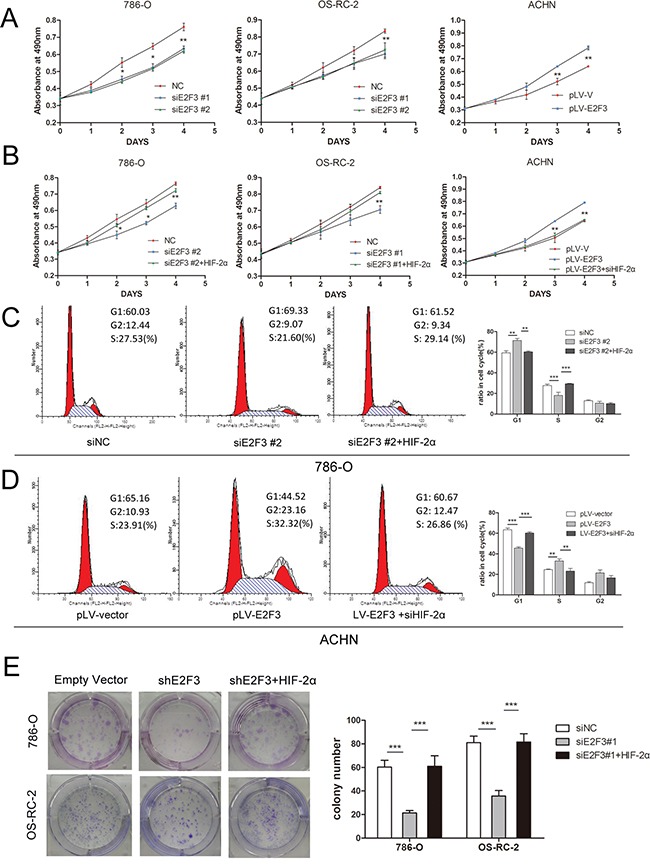
E2F3 facilitated cell proliferation and clonogenic survival through HIF-2α activation **A**. MTS assay showed E2F3 knockdown attenuated the proliferation velocity in 786-O and OS-RC-2 cells, whereas E2F3 overexpression promoted the tumor growth. Each experiment was performed in triplicate. **B**. Introducing HIF-2α into 786-O and OS-RC-2 cells partially reversed the effect of E2F3 on proliferation. Adversely, HIF-2α downregulation counteracted the effect of E2F3 on proliferation in ACHN. Data are shown as mean ± standard deviation (SD) (*p*<0.01). **C**. HIF-2α may combat the effect of G1 phase restoration by siE2F3 in 786-O cells (*p*<0.001). **D**. Overexpression of E2F3 accelerated the G1-S phase transition. The positive effect could be inhibited by siHIF-2α (*p*<0.001). **E**. Inhibitory effect of shE2F3 on colony formation could be reversed by HIF-2α. Representative experiments of three were with similar results. (*p<0.05, **p<0.01, ***p<0.001).

MTS assay was applied to validate whether the expression of E2F3 affected the proliferative ability of ccRCC cells through HIF-2α regulation. Compared with the control group, the growth curves demonstrated that the decreased expression of E2F3 significantly inhibited 786-O and OS-RC-2 cell growth (Figure 4A). Conversely, the overexpression of E2F3 accelerated ACHN cell growth. The addition of the lentiviral particles of HIF-2α into the siE2F3 group regained cell proliferative ability, whereas introducing siHIF-2α into the E2F3-expressed ACHN cells inhibited cell growth (Figure 4B). The possible mechanism behind the inhibitory effect of E2F3 knockdown was investigated using flow cytometry. The cell distribution in the cell cycle was analyzed by PI staining. As shown in Figure 4C, a statistically higher fraction of E2F3-knockdown 786-O cells were in the G0/G1 cell phases relative to their respective control groups. Similarly, a concomitant decrease in the S phase in these same compared cultures suggested that G0/G1 arrest occurred in the E2F3 knockdown cells. However, the cotransfection of HIF-2α with siE2F3 group may have prompted the cell cycle progression. E2F3 overexpression in ACHN cells drove the cell cycle from G1 phase into S phase while the HIF-2α knockdown restored the cell into G1 phase, suggesting that E2F3 may promote the proliferation of ccRCC cell lines through the activation of HIF-2α (Figure 4D). Results of the colony formation assay illustrated that compared with the empty vector group, shE2F3-transfected tumor cells impaired the colony-forming ability of 786-O and OS-RC-2 (Figure 4E). Given that the cell cycle is tightly modulated through a complex network of regulatory molecules including cyclin-dependent kinases (Cdks) and cyclins, the roles of CDK6 and CyclinD1 expression were examined. Supplementary Figure S1 showed that CDK6 and CyclinD1 were significantly downregulated in the knockdown of E2F3, 786-O, and OS-RC-2 cells accompanied with reduced HIF-2α expression. Adverse effects were seen in E2F3-overexpressed ACHN cells.

### HIF-2α overexpression is able to rescue the inhibiting capacity of cancer cell migration and invasion by E2F3 knockdown

**Figure 5:**
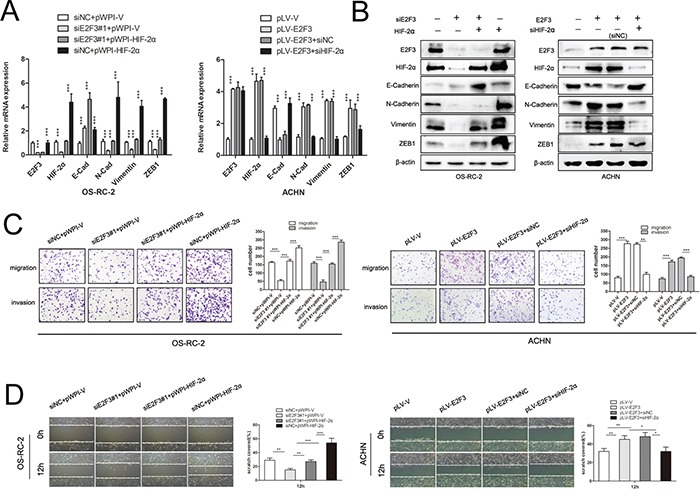
HIF-2α is critical for E2F3-mediated ccRCC migration and invasion **A**. E2F3, HIF-2α, and EMT markers’ expression mRNA levels were examined in ccRCC cell lines transfected with the vectors as indicated. **B**. E2F3, HIF-2α, and EMT markers’ expression in protein levels were evaluated in ccRCC cell lines transfected with the vectors as indicated. **C**. HIF-2α significantly increased migration and invasion of siE2F3 group. On the contrary, HIF-2α knockdown suppressed E2F3-mediated aggressiveness *in vitro* (*p*<0.001). **D**. For wound healing assay, the wound closure was photographed at different time points (0 and 12 h after scraping). In OS-RC-2 cells, E2F3 knockdown significantly decreased the number of viable cells (*p*<0.001), but cells regained migration capacity after HIF-2α transfection. In ACHN cells, HIF-2α knockdown suppressed the effect of E2F3 on cell viability (*p*<0.001). (*p<0.05, **p<0.01, ***p<0.001).

To understand the role of HIF-2α in E2F3-mediated migration, we ectopically expressed HIF-2α in E2F3-knockdown OS-RC-2 cells. Since HIF-2α is a transcription factor, the mRNA levels of E-Cadherin, N-Cadherin, Vimentin, and ZEB1 were subsequently examined after interference of E2F3 and HIF-2a (Figure 5A). As shown in Figure 5C, HIF-2α overexpression rescued the inhibitory effect of E2F3 knockdown on OS-RC-2 migration and invasion (*p*<0.001), suggesting that HIF-2α was involved in E2F3-mediated cell aggressiveness. Meanwhile, silencing HIF-2α significantly abrogated the invasive ability of E2F3 cell overexpression compared with the control group. Since E-Cadherin, N-Cadherin, Vimentin, and ZEB1 were considered as potential vital genes implying higher malignancy of tumor cells; Western blot analysis was performed to detect the protein level alteration. N-Cadherin, Vimentin, and ZEB1 were greatly downregulated after E2F3 knockdown, whereas E-Cadherin expression presented the adverse effect in OS-RC-2 (Figure 5B). After introducing HIF-2α, the invasion-attenuated cells re-expressed N-Cadherin, Vimentin, and ZEB1. Consequently, the overexpression of E2F3 significantly increased the HIF-2α and epithelial–mesenchymal transition (EMT)-related gene expression levels in ACHN. Then, the knockdown of HIF-2α counteracted the aggressiveness of cancer cells and changed the EMT-related gene expression levels as well. For the wound healing assay, the motility of tumor cells was assessed by intermediate empty space coverage 12 h after interference. In OS-RC-2, the E2F3 knockdown group had slower space coverage compared with the control group, whereas the HIF-2α transfection regained the migratory ability of siE2F3 group cells. In E2F3 lower-expressed cell line ACHN, the adoption of E2F3 plasmid speeded up cell migration while the HIF-2α knockdown impaired the migration ability (Figure 5D).

### E2F3 upregulates HIF-2α expression in mouse model

**Figure 6:**
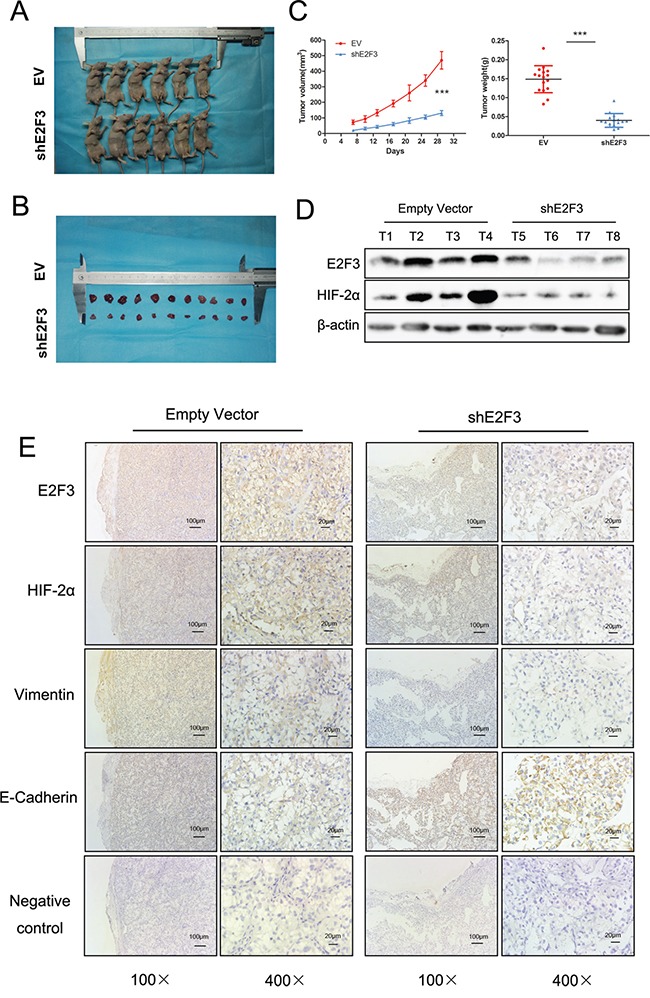
Attenuation of HIF-2α was found in stable downregulation of E2F3 expression of cancer cell lines *in vivo* **A**. The nude mice were terminated 29 days after subcutaneous injection of transfected shE2F3 or empty vector transfected cells. **B**. The subcutaneous tumors were resected and measured. **C**. The comparison of the tumor weight and volume in experimental and control group (*p*<0.001). **D**. Protein levels of E2F3 and HIF-2α were evaluated by Western blot analysis. **E**. Immunohistochemical staining showed the changes in EMT marker expression following stable downregulation of E2F3 expression. The effect of E2F3 on HIF-2α, E-Cadherin, and Vimentin expression was measured.

To confirm the role of E2F3 in renal cancer cell invasion, the tumor growth was monitored in 32 mice *in vivo*. Prior to injection, cells harboring either the empty vector or shE2F3 OS-RC-2 cells were harvested through trypsinization, washed in PBS, resuspended at 10^7^ cells/100 μl in a 1:1 solution of PBS/Matrigel, and injected subcutaneously into the left flank of the nude mice (Figure 6A). Tumors were then measured in three dimensions (a, b, c), and the volume to be calculated as abc × 0.52 (13). Twenty-nine days after the injection, the OS-RC-2 cells transfected with shE2F3 vector developed smaller tumors (0.148 ± 0.035 g weight, 470 ± 56 mm^3^ volume) than the control group (0.039 ± 0.018 g weight, 131 ± 16 mm^3^ volume) (*p*<0.001) (Figures 6B, 6C, and 6D). The mice were subsequently terminated for Western and immunohistochemistry analysis. The downregulation of E2F3 and HIF-2α protein in shE2F3 cells were confirmed by Western blot experiment (Figure 6D). Tumors were then characterized for the expression levels of several EMT-related proteins, such as E-Cadherin and Vimentin. Vimentin protein expression levels in shE2F3 group were significantly attenuated compared with control group in OS-RC-2 cells. E-Cadherin expression showed the adverse tendency. As expected, the expression of E2F3 in tumors with E2F3 knockdown was evidently lower than that observed in tumors from mice injected with control cells. The downregulated expression of HIF-2α in decreased E2F3 expression was further validated (Figure 6D). The results suggested that E2F3 is critical for carcinogenesis of ccRCC and HIF-2α may be the downstream gene of E2F3.

## DISCUSSION

Despite the surgical resection of ccRCC, chemotherapy and radiological therapy performed nearly invalid functions. Moreover, available efficient treatment was required apart from vascular endothelial growth factor (VEGF) pathway kinase inhibitors (sorafenib and sunitinib) and rapamycin (mTOR) blockers, which are now the first and second line therapies for late stage and metastatic ccRCC [17].

Mammalian E2F3 is a well-characterized transcription factor functioning in a cell cycle-dependent manner with specific binding to pRB, linking cell cycle proteins, such as cyclin-dependent kinases (CDKs) and cyclins [18]. Researchers have identified that E2F3 modulates different genes that play a crucial role in transcription, DNA synthesis, and signal transduction; E2F3 could also display apoptosis and drug resistance by initiating the downstream genes [19]. With strong oncogenic potential, E2F3 is regularly dysregulated or overexpressed in multiple cancers. As a master player, E2F3a was found to be essential in boosting the proliferation of ovarian cancer cells in response to the EGFR-driven mitogenic cell signal [20]. *in vitro* and *in vivo* studies adopted by Martinez demonstrated that E2F3 functions as a key regulator of DNA damage response and is pivotal for DNA damage-induced apoptosis [21]. Moreover, miR-200b interplayed with E2F3, contributing to the decrease in the chemical sensitivity of lung adenocarcinoma cells to docetaxel [22]. For prostate cancer, E2F3 may be an independent factor predicting overall and cause-specific survival [23]. Recently, studies revealed the involvement of E2F3 in cancer progression. Until now, the exact role of E2F3 in ccRCC has never been characterized.

As one of the most frequently investigated member of HIF-α family, HIF-2α enhanced the migratory and neoplastic capacities of hepatocellular carcinoma cells by promoting EMT [24]. In addition, hypoxia may trigger EMT process in low and highly aggressive pancreatic tumor cells with cancer stem cell characteristics [25]. HIF-2α triggered the alteration of cell morphology from epithelial to mesenchymal phenotype dominantly in CSC (high) cells [26]. SHARP1 has been reported to be a crucial regulator in the proteasomal degradation of HIF-1α for breast cancer progression without pVHL (von Hippel-Lindau tumor suppressor), hypoxia, and the ubiquitination mode [27]. Inactivation of pVHL plays an imperative role in the development of ccRCC, expressing either HIF-2α alone or both HIF-1α and HIF-2α [28]. Moreover, recent literature illustrated that HIF-2α, rather than HIF-1α, was the main tumor inducer in ccRCC [11]. Yet, relatively few researches have provided evidence on the crucial role of HIF-2α accumulation in cancer cells independent of hypoxia.

Protein translation is a biological process that occurs in the cytoplasm, whereas transcription occurs in the nucleus. Therefore, we hypothesized that both the absolute expression as well as the subcellular localization of E2F3 would predict clinicopathological features and overall survival in ccRCC. Clinical data analysis demonstrated the nuclear and cytoplasmic expression of E2F3 and its correlation with clinicopathological features. The variables age, size, gender, clinical stage, Fuhrman grade, MVI, and metastatic state were enrolled for further analysis. E2F3 expression was different in the nuclei and the cytoplasm. High cytoplasmic staining implicated large tumor size, high stage, high microvascular invasion, and high metastatic incidence. With close follow-up, subcellular localization of E2F3 signified different clinical outcomes; high E2F3 expression in the cytoplasm as well as low nuclear staining predicted short overall survival. The findings were in line with previous results of Nils Kroeger's study that tumors with high HIF-2α nuclear expression had smaller tumor sizes, lower T stages, and less advanced Fuhrman grades than those without [29]. Meanwhile, the upregulation of cytoplasmic HIF-2α expression was associated with cancer progression and worse survival outcome. Referring to the mechanism of HIF-2α's functions in the cytoplasm, Montagner M has illustrated that the HIF-2α–RBM4–eIF4E2 (eukaryotic translation initiation factor 4E) complex captured the 5′ cap and mRNAs targeted polysomes for active translation when assembled at the RNA hypoxia response element [27]. The propensity of E2F3 and HIF-2α in the same cellular location (cytoplasm) of ccRCC indicated that they may cooperate to facilitate tumor progression but the mechanism still needs to be explored.

Through direct binding to the promoter of a cluster of downstream genes, E2F3 was able to perform versatile functions. Stephen J et al. has identified that the Interleukin 6 receptor was a direct transcriptional target of E2F3 in prostate tumor-derived cells [30]. Lili He et al. reported that Aurora-A was a direct target of E2F3 during G2/M cell cycle progression for human ovarian cancer [31]. An interesting finding showing a significant association of the enhanced E2F3 expression with the HIF-2α expression motivated us to focus on the role of the relationship between E2F3 and HIF-2α in ccRCC development and progression. In the following study, we aim to investigate the mechanism of E2F3 in ccRCC progression and its relationship with HIF-2α. The clinical relevance of E2F3 and HIF-2α was confirmed by linear regression. Based on the putative binding site analysis, we elucidated that the overexpression of HIF-2α in ccRCC may be modulated in another pathway independent of the VHL-related axis. Chromatin immunoprecipitation assays showed the association of E2F3 with the HIF-2α promoters and luciferase experiments showed that HIF-2α promoters were responsive to E2F1 and E2F3. However, the E2F1 knockdown in the next step was not followed by attenuation of HIF-2α, indicating that the interaction of E2F1 and the promoter of HIF-2α was not a functional contact. As expected, transfection of siE2F3 inhibited HIF-2α expression.

HIF pathway is a positive regulator of tumor growth and malignancy. HIF activation correlates with metastasis in multiple tumors and can prompt metastasis through the tumor cell metastatic potential governing regulators, embracing lysyl oxidase (LOX), E-cadherin, CXCR4, and stromal-derived factor 1 (SDF-1)[32–34]. EMT is observed in a cluster of cancer cells undergoing phenotypic conversion for aggressiveness and metastasis. This process is defined by the depletion of epithelial cell junction markers such as E-cadherin, and the gain of mesenchymal proteins such as Vimentin ZEB1 and N-Cadherin [35]. In our study, as a member of a protein initiation complex, HIF-2α can rescue the migratory and invasive ability after transfection of siE2F3 in cell line. High levels of Vimentin, ZEB1, and N-Cadherin and low levels of E-cadherin were observed thereafter, suggesting that E2F3 may be a potential driver of EMT process through the activation of HIF-2α. Herein, we elucidated that cells expressing high levels of E2F3 could activate HIF-2α expression, thus leading to a series of EMT alterations. From the above results, we explored the effect of E2F3 in a mouse model. As we expected, tumors formed in the presence of E2F3-deficient cancer cells, displaying reduced weight and volume along with significantly less HIF-2α expression. On the sequential pathologic sections, several EMT-related gene expressions were also immunohistochemically examined.

An increasing amount of evidence suggested that E2F3 can trigger cancer cell growth and proliferation. AY Olsson et al. demonstrated that E2F3 levels can modify cellular proliferation rates in both bladder and prostate cancer [36]. MiR-195 can suppress the G1/S transition by blocking Rb-E2F signals through targeting E2F3, indicating that E2F3 affects cell cycle modulation of HCC [37]. MTS assay and cell cycle experiments were performed to demonstrate that E2F3 overexpression significantly enforced the proliferation and induced G1/S transition in cell line ACHN. Meanwhile, knockdown of E2F3 decreased proliferation velocity and led to G1 restoration in ccRCC cell line 786-O. Furthermore, we found that knockdown and overexpression of E2F3 caused a marked alteration in CDK6 and CyclinD1 expression, which are important factors in cell cycle regulation. Interestingly, HIF-2α overexpression was able to rescue the inhibition of cancer cell proliferation by E2F3 knockdown. Taken together, our data indicated that E2F3 triggered tumor cell growth and proliferation concomitantly with HIF-2α level. The above mentioned properties were completely consistent with our verification of E2F3 as a regulator of HIF-2α, which causes EMT and cancer promotion in ccRCC.

In summary, our data elucidated that cytoplasmic E2F3 was upregulated in high stage cancer. Moreover, *in vitro* overexpression of E2F3 cell migration and invasion, and metastasis in nude mice models were observed. HIF-2α overexpression in ccRCC is partially mediated by E2F3, which can directly bind to and transactivate the HIF-2α promoter. Our findings indicated that HIF-2α was a novel direct E2F3 target gene that promotes ccRCC carcinogenesis and progression. In conclusion, E2F3 overexpression may transcriptionally upregulate HIF-2α, thus inducing higher cancer malignancy in ccRCC by boosting proliferation, migration, and invasion capacity of cancer cells. E-Cadherin, Vimentin, ZEB1, and N-Cadherin were introduced as the indicators in cancer progression, and their fluctuations were associated with the E2F3 and HIF-2α change.

Finally, the E2F3- HIF-2α axis could be an important target for cancer intervention. Further studies are needed to identify whether disrupting the E2F3-HIF-2α interaction could be a promising way of combating cancer progression and metastasis of ccRCC tissues.

## MATERIALS AND METHODS

### Ethics statement

Written informed consent was obtained from all patients before sample collection and the study was admitted by the Protection of Human Subjects Committee of Chinese People's Liberation Army (PLA) General Hospital. All animal experiments were carried out following protocols approved by the Institutional Animal Care and Use Committee of Chinese PLA General Hospital.

### Patients and specimen collection

The study cohort consisted of 112 ccRCC patients who were admitted at the Chinese PLA General Hospital's Department of Urology from January 2005 to December 2010. The cancer tissues were collected and immunohistochemically analyzed. All tissue samples have been clinically and pathologically confirmed to be of the clear cell type and were staged according to the 2011 Union for International Cancer Control TNM classification of malignant tumors. The Fuhrman nuclear grading system was used to determine the nuclear grade. Microvascular invasion (MVI) displayed small vessel invasion which indicated tumor aggressiveness.

### Cell line and cell culture

The ccRCC cell lines ACHN, 786-O, OS-RC-2, and Caki-2 were preserved in our laboratory. The cells were cultured in Dulbecco's modified Eagle's medium (HyClone), McCoy's 5A Medium (HyClone), DMEM/F12 (HyClone) with 10% fetal bovine serum (Gibco, USA), penicillin (100 U/ml), and streptomycin (100 U/ml) according to the American Type Culture Collection. All cells were cultivated in a sterile incubator maintained at 37 °C with 5% CO_2_.

### Immunohistochemistry

Immunohistochemistry was performed on renal cancer specimens as previously described. The evaluation of E2F3 and HIF-2α protein expression was independently and blindly analyzed by two observers (Y. F and YX. Y). According to the previous study, staining intensity was scored 0 (negative), 1 (weak), 2 (moderate), and 3 (strong). For convenience in the Chi-square test, the final score was determined by multiplying the intensity scores with staining extent, and the results were ranged from 0–9. Final scores (intensity score × percentage score) less than 2 were considered as negative staining, 2–3 as weak staining (+), 4–6 as moderate staining (++), and >6 as strong staining (+++)[38].

### Western blot assay

The total protein of the tumor cells was obtained using RIPA lysis buffer (Santa Cruz) mixed with proteinase inhibitors (Roche Applied Science). Western blot assays were performed on RCC lines and samples as previously described. Blots were incubated with primary antibodies at 4 °C overnight; the information on all primary antibodies was listed in Table S1. In all specimens, goat anti-mouse IgG-HRP and goat anti-rabbit IgG-HRP (ZSGB-BIO) were used as the secondary antibody at a dilution of 1:8000. Specific proteins were visualized using an enhanced chemiluminescence detection reagent (Pierce) and exposed to X-ray film (Kodak, Rochester, NY).

### siRNA and plasmid constructs

Small interfering RNAs (siRNAs) against E2F3, E2F1, E2F2, and HIF-2α were designed and synthesized from GenePharma (Shanghai, China) (Table S1). The fragment of E2F3 coding sequence was inserted into the lentiviral vector pLV-EGFP-(2A)Puro (Inovogen Tech. Co.). XbaI and EcoRI were used to generate pLV-EGFP-E2F3, and the desired sequence was identified through DNA sequencing. Lentivirus-encoding DNA was packaged as previously described [39]. To achieve the shE2F3 plasmid, siE2F3#1 sequence was cloned into pLVshRNA-EGFP-(2A)Puro plasmid. After transfection of the above plasmid, E2F3-overexpressed and knockdown cancer cells were established (Puromycin selection). Post-transfection of E2F3 expression was evaluated using real-time PCR (RT-PCR) and Western blot analysis. Results of transfection efficiency are shown in Figure S2. Genomic DNA for the cloning of HIF-2α promoters was extracted from 293T cells using standard protocols. Primers spanning 1.8kb of the promoter HIF-2α were used to amplify the fragment. The fragments were then subcloned into pGL3-basic luciferase vector (Promega) with the enzymatic activation of Kpn1 and Hind III. Gibson Assembly box was applied to construct mutation plasmid [40].

### Cell immunofluorescence

The cells of different groups were seeded and grown on glass slides 24 h before the experiment. After fixation with 4% paraformaldehyde-PBS for 15 min, cells were washed once in PBS and then permeabilized with 0.5% Triton X-100. Then, 3% bovine serum albumin was introduced to block the cells for 30 min. Coverslips were stained with primary antibody (E2F3 and HIF-2α) at 37 °C for 1 h and incubated with fluorescein isothiocyanate (FITC)-conjugated goat anti-rabbit IgG and Rhodamin conjugated goat anti-mouse IgG as secondary antibody (ZSGB-BIO), respectively. Application of 0.2 mg/mL DAPI was done for nuclei staining. Slides were viewed using Olympic confocal microscopy. Pictures were merged by OLYMPUS Fluoview FV1000 (version 1.6).

### Luciferase assay

Genomic DNA fragments of the human HIF-2α gene, spanning from +1 to -1800 relative to the transcription initiation site, were generated by PCR and inserted into pGL3-Basic vectors (denoted as pGL3–HIF-2α). All constructs were confirmed by DNA sequencing. Cells were plated at a density of 5 × 10^5^ cells/well in 6-well plates prior to transfection. 293T cells were transfected with 0.5 μg of HIF-2α reporters along with 0.5 μg, 1 μg and 2 μg E2F3 expression vectors using lipofectamin 2000. Cotransfection was done with pRL construct containing *Renilla reniformis* luciferase gene, which was used as normalizing control. Luciferase assays were performed using Dual Luciferase Assay System (Promega). Relative luciferase activity was defined as the ratio of firefly luciferase activity to *R*. *reniformis* luciferase activity. Error bars represented standard deviation of the three experiments.

### Chromatin immunoprecipitation (ChIP) assays

Chromatin immunoprecipitation (ChIP) assays were performed on OS-RC-2 and 786-O cells using antibody for E2F3, with anti-rabbit IgG as the negative control. The interaction with predicted promoters was detected using real-time PCR with primer sequences detailed in Supplemental Table S1. The whole procedure was performed using the ChIP assay kit (Upstate EZ-CHIP, 17-371) following the manufacturer's instructions. Briefly, the cells were cross-linked with 1% formaldehyde for 10 min at room temperature. DNA-protein immunocomplexes were immunoprecipitated using E2F3 antibody, normal rabbit IgG, and positive control. Ips and inputs were incubated at 65 °C overnight to reverse the DNA-protein crosslinks. The purified DNA was subjected to quantitative real-time PCR.

### MTS assay and colony formation

Cell proliferative ability was measured by absorbance using the MTS assay. The cells that were transfected with siRNA duplex or viral particles were seeded into 96-well plates (1000 cells/well) and cultured with 200 μl of 10% FBS/medium at 37 °C in a 5% CO_2_ incubator. At the scheduled time points (24, 48, 72, and 96 h), 20 μl of CellTiter 96 Aqueous One Solution (Promega, Madison, WI) was added to each well and incubated for 1 h at 37 °C. Absorbance was measured at 490 nm using an automatic enzyme-linked immunosorbent assay reader (BioTek Instruments), which was used according to manufacturer's instruction. For the anchorage-dependent growth assay, 786-O and OS-RC-2 cells were separately seeded in six-well culture plates 48 h after transfection at a density of 1×10^3^ cells per well. Colony numbers were counted after the cells were fixed with methanol and stained in 0.2% crystal violet at day 14.

### Cell cycle analysis

After transfection for 48 h, the 786-O and ACHN cells were collected, washed with PBS, and fixed in 70% ice-cold ethanol overnight. The cells were then treated with propidium iodide (Beyotime, Shanghai, China) according to the manufacturer's instructions. FACS-Calibur (BD Biosciences) was used to verify the cell cycle change. Each experiment was performed in triplicate and repeated three times.

### Cell migration and invasion assay

Cell migration and invasion assays were performed in 24-well plates using Boyden chambers that contained Transwell (Corning, NY) membrane filter inserts with a pore size of 8 μm. For the invasion assay, the membrane undersurface was coated with 50 mL of 1:3 diluted Matrigel (BD Biosciences), which had solidified at 37 °C. Approximately 2 × 10^4^ cells in 200 mL of culture medium supplemented with 1% FBS was seeded into the upper chamber, whereas the lower chamber was filled with the high concentration medium (10% FBS). After 4 (migration) or 8 (invasion) h at 37 °C, invading and migrating cells were fixed and stained with 0.1% crystal violet. The lower surfaces of the membranes were photographed at 100× magnification and five random fields were photographed from each chamber to determine the migration. All assays were performed independently three times. The subsequent staining and observation procedures were identical to those of the migration assays.

### Wound healing assay

For the wound healing assay, OS-RC-2 and ACHN were seeded on 6-well plates with medium containing 1% FBS. Confluent monolayer cells were scratched using a sterile 200 μL pipette. Pictures of the wound were taken at different time points (0 and 12 h after scratching) and the coverage of the scratching area was measured at three different positions for each replicate. The above experiments were performed in triplicate.

### *In vivo* tumor growth assay

All experimental procedures involving animals were performed according to guidelines for the care and use of laboratory animals and institutional ethical guidelines for animal experiments. OS-RC-2/empty vector or OS-RC-2/shE2F3 cells (10^7^ cells/100 μl in a 1:1 solution of PBS/Matrigel) were implanted subcutaneously into the left armpit of 4–5-week-old male nude mice (16 mice per group). Tumor volume was measured in three dimensions (a, b, c) every one week using calipers and calculated as abc × 0.52.

### Statistical analysis

Statistical analysis was performed using SPSS 18.0 (SPSS Inc., Chicago, IL, USA) and Student's t-test was used to compare mean values for paired data. Normally distributed data are expressed as mean ± standard deviation (SD) and comparisons were performed using Student's t-test. Abnormally distributed data and comparisons were performed using Mann-Whitney U tests. The categorical data was analyzed using either Fisher's exact or the Chi-Square method. The Kaplan-Meier and log-rank tests were used for the overall survival analysis.

## SUPPLEMENTARY MATERIALS FIGURES AND TABLE


