# Supplementary material for: E2F3 upregulation promotes tumor malignancy through the transcriptional activation of HIF-2α in clear cell renal cell carcinoma
Source: Oncotarget. 2016 Jul 13;8(33):54021–36. doi: 10.18632/oncotarget.10568 (PMC5589559; doi:10.18632/oncotarget.10568)
Supplement: Supplementary file 1 [file oncotarget-08-54021-s001.pdf]

# E2F3 upregulation promotes tumor malignancy through the transcriptional activation of HIF-2α in clear cell renal cell carcinoma

## SUPPLEMENTARY FIGURES AND TABLE

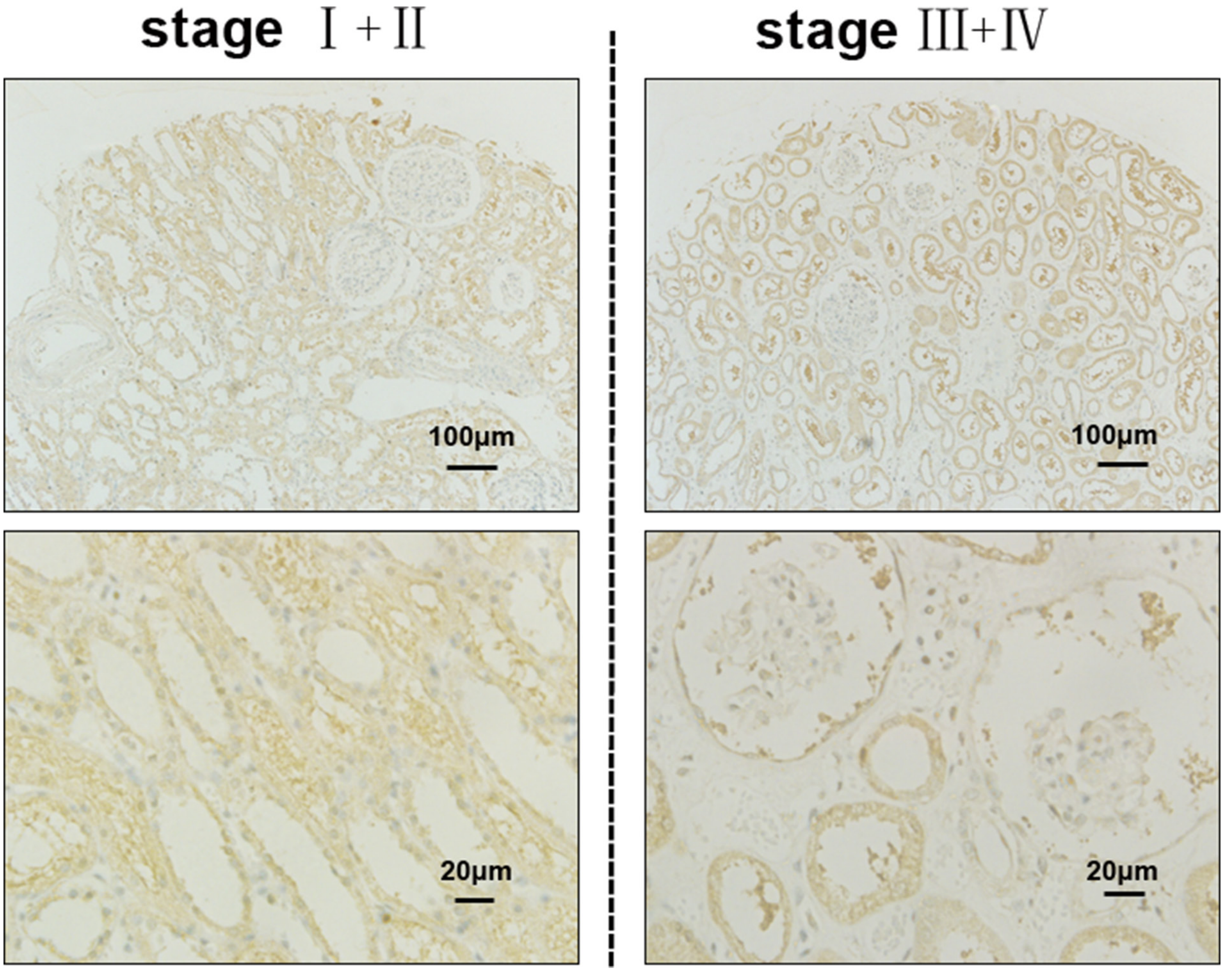


**Supplementary Figure S1: The E2F3 staining in normal kidney tissues for different stages of renal cancer.**

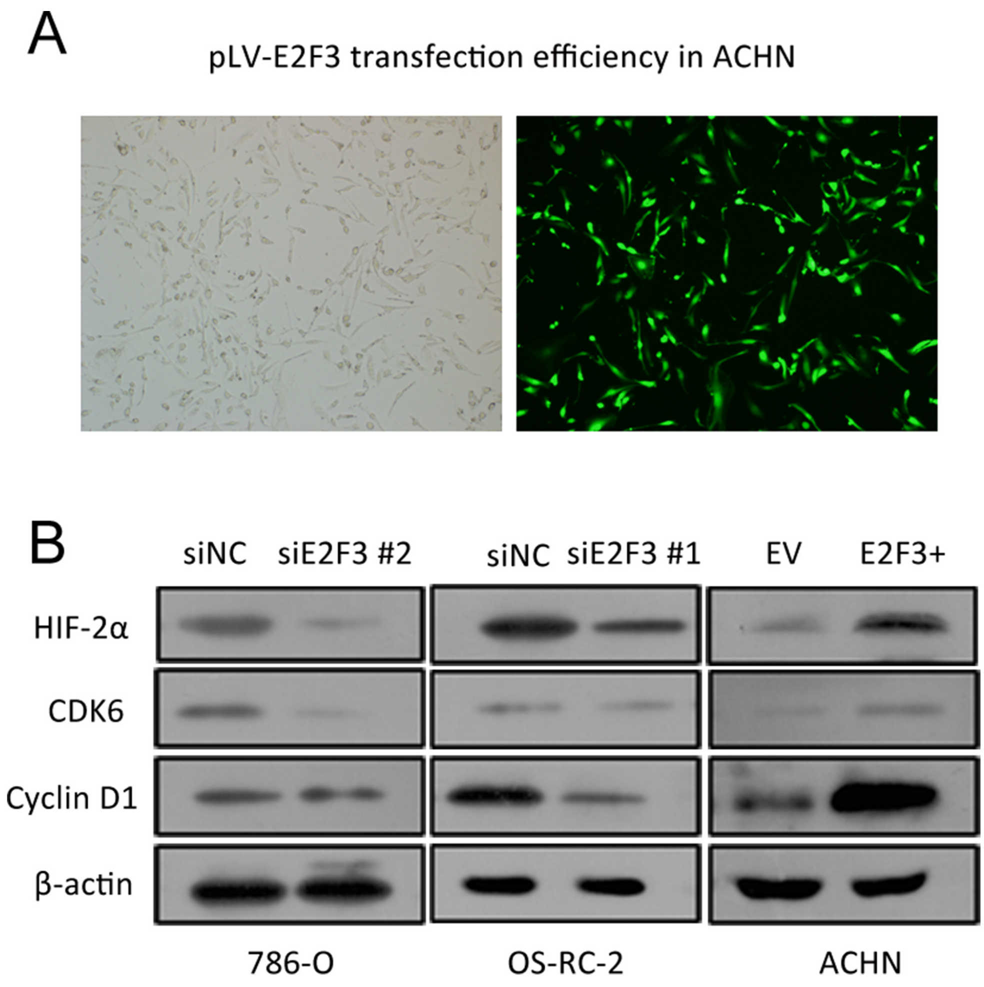


**Supplementary Figure S2: A.** pLV-E2F3 transfection efficiency was identified in ACHN cells. **B.** The effects of E2F3 knockdown and overexpression on HIF-2α expression in cell lines were examined by Western blot analysis. CDK6 and Cyclin D1 proteins were tested in parallel.

**Supplementary Table S1: Antibodies and primers**

| **Antibodies** | | | | |
|---|---|---|---|---|
| **Name** | **Manufacturer** | **Number** | **Type** | **Usage** |
| E2F3 | SANTA CRUZ | sc-879 | Polyclonal | WB,IHC,IF,CHIP |
| E2F1 | SANTA CRUZ | sc-193 | Polyclonal | WB |
| E2F2 | SANTA CRUZ | sc-632 | Polyclonal | WB |
| HIF-2α | NOVUS BIOLOGICALS | NB100-132 | Monoclonal | WB,IHC,IF |
| β-actin | ZSGB-BIO | TA-09 | Monoclonal | WB |
| GAPDH | ZSGB-BIO | TA-08 | Monoclonal | WB |
| E-Cadherin | Cell Signaling Technology | #3195 | Monoclonal | WB,IHC |
| Vimentin | Cell Signaling Technology | #5741 | Monoclonal | WB,IHC |
| ZEB1 | Cell Signaling Technology | #3396 | Monoclonal | WB |
| N-Cadherin | Abcam | Ab76057 | Polyclonal | WB |
| Cyclin D1 | EPITOMICS | #2261-1 | Monoclonal | WB |
| CDK6 | EPITOMICS | #3524-1 | Monoclonal | WB |
| **siRNAs** | **sequences (5'-3')** | | | |
| E2F3 #1 | GCUCACCAAGAAGUUCAUUTT/AAUGAACUUCUUGGUGAGCTT | | | |
| E2F3 #2 | GCAUCCACCUCAUUAAGAATT/UUCUUAAUGAGGUGGAUGCTT | | | |
| E2F3 #3 | GCGAUUGCUCAGUUUCUAUTT/AUAGAAACUGAGCAAUCGCTT | | | |
| E2F1 | GCGCAUCUAUGACAUCACCTT/GGUGAUGUCAUAGAUGCGCTT | | | |
| E2F2 | CCUACUACACACCGCUGUATT/UACAGCGGUGUGUAGUAGGTT | | | |
| HIF-2α | CAGCAUCUUUGAUAGCAGUTT/ACUGCUAUCAAAGAUGCUGTT | | | |
| NC | UUCUCCGAACGUGUCACGUTT/ACGUGACACGUUCGGAGAATT | | | |
| **Primer sequences (5'-3')** | | | | |
| E2F3 | F:AACAACCAAGACCACAATG | R:AATCGCTATGTCCTGAGTT | | |
| E2F2 | F:AAGTGCATCAGAGTGGAT | R:AGTGTCATACCGAGTCTTC | | |
| E2F1 | F:CAGAGCAGATGGTTATGG | R: CTGAAAGTTCTCCGAAGA | | |
| HIF-2α | F:TGGCGACATGATCTTTCTGTCA | R:ATGGTCGCAGGGATGAGTGA | | |
| PPIA | F:ATGGTCAACCCCACCGTGT | R:TCTGCTGTCTTTGG GACCTTGTC | | |
| HIF-2α-P*(1) -P*(2)-P*(1) | F: CAAGTTGGGCAGATCACTTG | R: ACTCTGTCGCCCAGGCT | | |
| HIF-2α-P*(2) | F: AGCCTGGGCGACAGAGTG | R: CTACAGAAGCGATTTGAAGAGA | | |
| HIF-2α-P*(3) | F: ATTATCCCCACCTGGCC | R: AGAGGCAGCCTGCGCA | | |

WB: Western boltting; IHC: Immunohistochemistry; IF:Immunofluorescence; siRNA: Small interfering RNA; P*: Promoter
